# Sugar Overconsumption during Adolescence Selectively Alters Motivation and Reward Function in Adult Rats

**DOI:** 10.1371/journal.pone.0009296

**Published:** 2010-02-19

**Authors:** Leandro F. Vendruscolo, Aliou B. Gueye, Muriel Darnaudéry, Serge H. Ahmed, Martine Cador

**Affiliations:** 1 CNRS-UMR5227, Université de Bordeaux, Bordeaux, France; 2 CNRS-UMR5226, Université de Bordeaux, Bordeaux, France; L'université Pierre et Marie Curie, France

## Abstract

**Background:**

There has been a dramatic escalation in sugar intake in the last few decades, most strikingly observed in the adolescent population. Sugar overconsumption has been associated with several adverse health consequences, including obesity and diabetes. Very little is known, however, about the impact of sugar overconsumption on mental health in general, and on reward-related behavioral disorders in particular. This study examined in rats the effects of unlimited access to sucrose during adolescence on the motivation for natural and pharmacological rewards in adulthood.

**Methodology/Principal Findings:**

Adolescent rats had free access to 5% sucrose or water from postnatal day 30 to 46. The control group had access to water only. In adulthood, rats were tested for self-administration of saccharin (sweet), maltodextrin (non-sweet), and cocaine (a potent drug of abuse) using fixed- and progressive-ratio schedules, and a concentration-response curve for each substance. Adult rats, exposed or not exposed to sucrose, were tested for saccharin self-administration later in life to verify the specificity of adolescence for the sugar effects. Sugar overconsumption during adolescence, but not during adulthood, reduced the subsequent motivation for saccharin and maltodextrin, but not cocaine. This selective decrease in motivation is more likely due to changes in brain reward processing than changes in gustatory perception.

**Conclusions/Significance:**

Sugar overconsumption induces a developmental stage-specific chronic depression in reward processing that may contribute to an increase in the vulnerability to reward-related psychiatric disorders.

## Introduction

Throughout the course of human evolution resources have been scarce and thus evolution has fostered a reward-based drive to obtain them. Due to the increased accessibility of food, feeding habits have dramatically changed in just a few centuries or even decades [1], [2]. Notably, the consumption of sugar has escalated in modern societies. Once considered a luxury, refined sugar (e.g., sucrose) has become cheap and enormously popular [1]. Today, the average daily sugar intake exceeds the level recommended by the World Health Organization and the Food and Agriculture Organization of the United Nations (WHO/FAO), which indicates that sugar intake should be limited to less than 10% of caloric intake [3]. An important contributor to this sugar overconsumption is the widespread availability of drinks containing sugar (e.g., soft drinks) at relatively low cost [2], [4], [5]. For instance, data from the U.S. estimates a per capita increase in the consumption of sweetened drinks of around 500% over the past 60 years [2]. Of particular concern is the fact that adolescent sugar intake is higher than any other age group [5], [6]. From a developmental standpoint, this is a period characterized by marked physical and emotional maturational changes and therefore is highly susceptible to environmental influences [7]–[9]. These findings have stimulated extensive research on the nutritional and physiological consequences (e.g., dental decay, weight gain, diabetes, metabolic syndrome, cardiovascular diseases, etc.) associated with sugar overconsumption [2], [6], [10], [11]. However, the potential impact of this deregulation of diet on the brain reward and motivational systems has rarely been directly addressed [12], [13].

A sweet taste produces a sensation of intense reward [14] that, in certain circumstances, exceeds those associated with drugs of abuse [15], [16]. Studies in humans and experimental animals suggest that sugar overconsumption may produce neurobiological and behavioral alterations resembling drug addiction [17]–[19]. More specifically, the intake of sugar or sweetened foods may elicit food-seeking behaviors [14], [20], [21], bingeing [22], escalation of intake [20], [23], sensitization, cross-sensitization to psychostimulants and opioids [24], and even withdrawal symptoms [25]. Given that exposure to drugs of abuse, particularly during critical periods of brain development (e.g., adolescence), produces enduring changes in the brain reward system and behavior [26]–[29], one could wonder whether, and to what extent, overconsumption of sweetened drinks earlier in life might produce similar persistent neurobehavioral alterations in adulthood.

Herein, we investigated the effects of unlimited access to sucrose solution during adolescence on the intake of and motivation for natural (sweet and non-sweet solutions) and pharmacological (cocaine) rewards in adult rats. We used cocaine because studies indicate that the motivational properties of sugar and cocaine may share similar neurobiological substrates (e.g., dopamine system) [30], [31]. We targeted adolescence because profound developmental changes in the brain reward system occur during this phase [7]–[9] and, concomitantly, the consumption of sugar in humans is highest during this period of life [5], [6]. Furthermore, we wanted to evaluate whether the behavioral changes produced by sugar overconsumption were specific to alterations that occur during adolescence, or whether sugar overconsumption by adult rats would also produce behavioral changes later in adulthood.

## Results

### Consummatory Behavior and Body Weight during Adolescence

Rats having access to sucrose drank more of this solution (59.8±6.2 ml/day; t_14_ = 6.1, p<0.0001) and less water (3.3±0.7 ml/day; t_14_ = 16.4, p<0.0001) compared to water consumed by control rats (21.5±0.8 ml/day). Overconsumption of sucrose led to a 10.8% reduction in food intake (17.3±0.4 g/day vs. 19.4±0.3 g/day by controls; t_14_ = 3.8, p<0.01). However, the total energy intake was 10.7% higher in the group having access to sucrose solution (62.2±1.7 Kcal/day; t_14_ = 3.2, p<0.01) compared to the controls (56.2±0.8 Kcal/day). These results confirm that caloric compensation is less adequate when liquid calories are ingested [32]. In relation to the total daily energy intake, 20.6% and 2% of calories were derived from sugar for the sucrose and control groups, respectively. Despite differences in the calorie intake during the period of sugar exposure, body weight was similar between the experimental groups (sucrose group: P30, 84.4±1.4 g and P46, 174.4±3.6 g; water group: P30, 84.1±2.2 g and P46, 177.0±3.4 g).

### Behavioral Testing in Adulthood

Adult rats, exposed or not exposed to sucrose during adolescence, were trained to lever press for saccharin, maltodextrin or cocaine using fixed-ratio (FR) 1 (measure of intake) and FR5 (measure of intake/motivation) schedules. This procedure was followed by progressive-ratio (PR; measure of motivation) and concentration-response (measure of reward efficacy and gustatory/pharmacological sensitivity) tests.

### Sugar Overconsumption during Adolescence Reduces the Motivation for Sweet Solution in Adulthood

**Figure 1:**
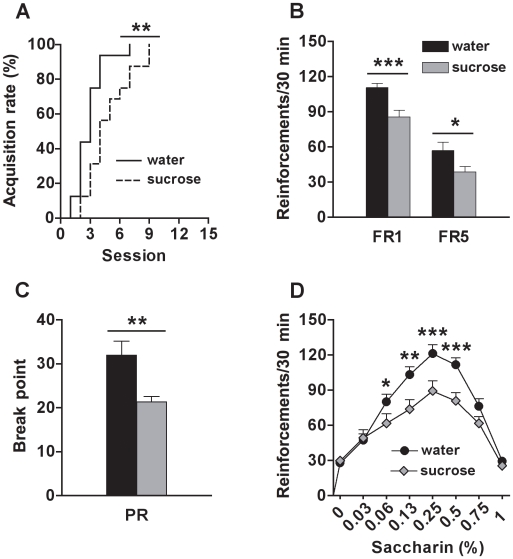
Motivation for saccharin (sweet) in adult rats exposed to sucrose or water during adolescence. (A) Percentage of rats reaching the acquisition criterion (>50 reinforcements for 2 consecutive sessions) for saccharin (0.13%, w/v) self-administration; (B) Number of saccharin (0.13%, w/v) reinforcements earned on fixed-ratio 1 (FR1) and fixed-ratio 5 (FR5) schedules; (C) Breakpoint reached during the progressive-ratio (PR) test; and (D) Number of reinforcements earned (FR5) at different saccharin concentrations (in %, w/v), by adult rats exposed to sucrose (N = 15) or water (N = 16) during adolescence. *, **, *** Indicate significant group differences (p<0.05, p<0.01 and p<0.001, respectively).

Sucrose-exposed rats met the acquisition criterion for saccharin self-administration later than control rats (χ^2^ = 7.2, *df* = 1, p<0.01) (Figure 1A). During the FR1 and FR5 sessions, sucrose-exposed rats earned less saccharin reinforcements than control rats (group effect: FR1, F_(1, 30)_ = 13.7, p<0.001; FR5, F_(1, 30)_ = 4.7, p<0.05) (Figure 1B). The level of response increased across sessions (session effect: FR1, F_(14, 420)_ = 70.6, p<0.0001; FR5, F_(9, 270)_ = 11.7, p<0.0001), but similarly for both groups (group x session interaction: n.s.). Moreover, sucrose-exposed rats were less motivated for saccharin, as these animals reached lower breakpoints during the PR test, compared to the controls (group effect: F_(1, 29)_ = 9.0, p<0.01) (Figure 1C). No significant differences were found for the session factor (n.s.) and for the interaction between group and session factors (n.s.). During the concentration-response test, a bell-shaped curve was observed, with lower response levels at higher and lower concentrations of saccharin, and higher response levels for intermediate concentrations (concentration effect: F_(7, 203)_ = 88.7, p<0.0001). A group effect (F_(1, 29)_ = 5.9, p<0.05) and a concentration x group interaction (F_(7, 203)_ = 5.1, p<0.0001) were also revealed by the ANOVA. The post-hoc comparisons indicated that sucrose-exposed rats showed lower response levels for saccharin at 0.06% (p<0.05), 0.13% (p<0.01), 0.25% (p<0.001) and 0.5% (p<0.001) compared to the controls (Figure 1D).

### Sugar Overconsumption during Adolescence Reduces the Motivation for Non-Sweet Solution in Adulthood

**Figure 2:**
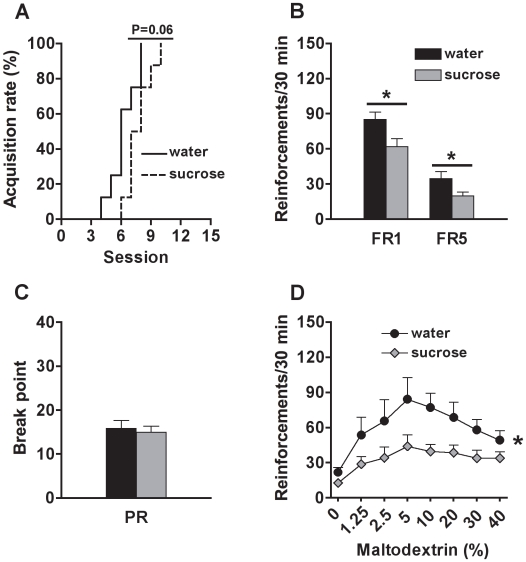
Motivation for maltodextrin (non-sweet) in adult rats exposed to sucrose or water during adolescence. (A) Percentage of rats reaching the acquisition criterion (>50 reinforcements for 2 consecutive sessions) for maltodextrin (5%, w/v) self-administration; (B) Number of maltodextrin (5%, w/v) reinforcements earned on fixed-ratio 1 (FR1) and fixed-ratio 5 (FR5) schedules; (C) Breakpoint reached during the progressive-ratio (PR) test; and (D) Number of reinforcements earned (FR5) at different maltodextrin concentrations (in %, w/v), by adult rats exposed to sucrose (N = 8) or water (N = 8) during adolescence. * Indicates significant group differences (p<0.05).

A non-significant marginal effect was detected for the acquisition rate (χ^2^ = 3.6, *df* = 1, p = 0.058), with sucrose-exposed rats acquiring maltodextrin self-administration at a slightly slower rate than control rats (Figure 2A). During the FR1 and FR5 tests, sucrose-exposed rats earned less maltodextrin reinforcements than control rats (group effect: FR1, F_(1, 14)_ = 6.2, p<0.05; FR5, F_(1, 14)_ = 5.3, p<0.05) (Figure 2B). The level of response increased across the sessions (session effect: FR1, F_(14, 196)_ = 55.6, p<0.0001; FR5, F_(9, 126)_ = 3.9, p<0.001), independently of groups (group x session interaction: n.s.). During the PR test, similar breakpoints were reached for the experimental groups (group effect: n.s.) (Figure 2C). The breakpoints varied among the sessions (session effect: F_(4, 56)_ = 3.1, p<0.02), independently of group (group x session interaction: n.s.). For the concentration-response curve, the ANOVA revealed an effect of group (F_(1, 14)_ = 4.6, p<0.05) and of concentration (F_(7, 98)_ = 10.0, p<0.0001); however, the interaction between these factors was not statistically significant (n.s.). These data indicate that sucrose-exposed rats showed overall lower response levels, regardless of maltodextrin concentration, compared to control rats (Figure 2D).

### Sugar Overconsumption during Adolescence Does Not Alter the Motivation for Cocaine in Adulthood

**Figure 3:**
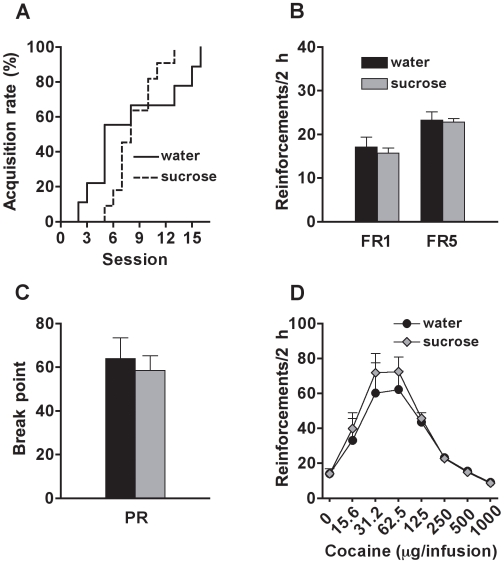
Motivation for cocaine in adult rats exposed to sucrose or water during adolescence. (A) Percentage of rats reaching the acquisition criterion (>15 reinforcements for 2 consecutive sessions) for cocaine (250 µg/infusion) self-administration; (B) Number of cocaine (250 µg/infusion) reinforcements earned on fixed-ratio 1 (FR1) and fixed-ratio 5 (FR5) schedules; (C) Breakpoint reached during the progressive-ratio (PR) test; and (D) Number of reinforcements earned (FR5) at different cocaine concentrations (in µg/infusion), by adult rats exposed to sucrose (N = 10) or water (N = 9) during adolescence.

The statistical analysis indicated that sucrose-exposed and control rats similarly acquired cocaine self-administration (n.s.) (Figure 3A). Moreover, they showed similar levels of cocaine intake during the FR1 (group effect: n.s.) and FR5 (group effect: n.s.) tests (Figure 3B). The response for cocaine increased across sessions for both FR1 (F_(16, 288)_ = 83.8, p<0.0001) and FR5 (F_(5, 90)_ = 3.1, p<0.05), independently of groups (group x session interaction for FR1 and FR5: n.s.). During the PR test the breakpoint did not differ between the experimental groups (group effect: n.s.) (Figure 3C) and response levels were stable across sessions (session effect: n.s.) for both groups (group vs. session: n.s.). For the concentration response, a bell-shaped curve (concentration effect: F_(7, 126)_ = 31.8, p<0.0001) was observed, with no difference between groups (group effect and group x concentration interaction: n.s.) (Figure 3D).

### Sugar Overconsumption During Adulthood Does Not Alter the Motivation for Sweet Solution Later in Life

The pattern of consumption displayed by adult rats during the period of sucrose access was very similar to that observed for adolescent rats. Adult rats having access to sucrose drank more of this solution (88.6±14.5 ml/day; t_14_ = 4.1, p<0.001) and less water (2.8±0.6 ml/day; t_14_ = 24.6, p<0.0001) compared to water consumption by control rats (28.2±0.8 ml/day). Overconsumption of sucrose led to a 13.8% reduction in food intake (19.3±0.5 g/day vs. 22.4±0.7 g/day by controls; t_14_ = 3.5, p<0.01). However, the total energy intake was 13.2% higher in the group having access to sucrose solution (73.6±2.7 Kcal/day; t_14_ = 2.5, p<0.05) compared to the controls (65.0±2.2 Kcal/day). Regarding the total daily energy intake, 25.5% and 2% of calories were derived from sugar for sucrose and control groups, respectively. Body weight was similar for the experimental groups (sucrose group: P60, 243.6±5.6 g and P76, 305.5±8.0 g; water group: P60, 242.3±4.5 g and P76, 302.4±8.2 g).

**Figure 4:**
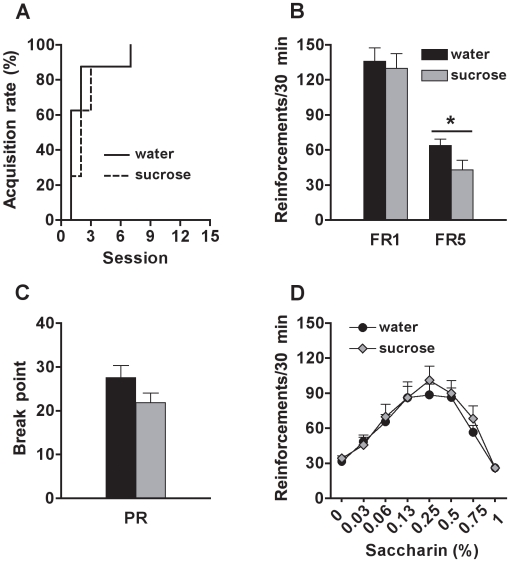
Motivation for saccharin (sweet) in rats exposed to sucrose or water during adulthood. (A) Percentage of rats reaching the acquisition criterion (>50 reinforcements for 2 consecutive sessions) for saccharin (0.13%, w/v) self-administration; (B) Number of saccharin (0.13%, w/v) reinforcements earned on fixed-ratio 1 (FR1) and fixed-ratio 5 (FR5) schedules; (C) Breakpoint reached during the progressive-ratio (PR) test; and (D) Number of reinforcements earned (FR5) at different saccharin concentrations (in %, w/v), by rats exposed to sucrose (N = 8) or water (N = 8) during adulthood. * Indicates significant group differences (p<0.05).

Sucrose-exposed and control rats similarly acquired saccharin self-administration (group effect: n.s.) (Figure 4A). During the FR1 sessions, rats progressively increased their response (session effect: F_(14, 196)_ = 22.2, p<0.0001) and the experimental groups did not differ statistically (group effect and group x session interaction: n.s.) (Figure 4B). In contrast, sucrose-exposed rats showed lower levels of saccharin self-administration during the FR5 test compared to control rats (group effect: F_(1, 14)_ = 4.6, p<0.05) (Figure 4B). Saccharin self-administration increased across sessions (session effect: F_(9, 126)_ = 14.2, p<0.0001), independently of group (group x session: n.s.). During the PR test, rats of both groups reached similar breakpoints and the level of response stable across sessions (group effect, session effect and group x session interaction: n.s.) (Figure 4C). Furthermore, the experimental groups showed similar saccharin self-administration during the concentration-response test (group effect and group x concentration interaction: n.s.), with the response of both groups following a bell-shaped curve (concentration effect: F_(7, 98)_ = 39.7, p<0.0001) (Figure 4D).

### Saccharin Overconsumption during Adolescence Reduces the Motivation for Sweet Solution in Adulthood

In order to determine whether the reported effects of sucrose were due to its sweet taste or caloric load, adolescent rats were given continuous access, in free choice with water, to a saccharin solution (0.25%, w/v), instead of 5% sucrose, for 16 days (N = 8). This concentration of saccharin is equally preferred (isopalatable) in comparison to 5% sucrose (unpublished data). The control group had access to water only (N = 8).

Rats having access to saccharin drank more of this solution (41.0±7.1 ml/day; t_14_ = 2.7, p<0.05) and less water (3.9±1.4 ml/day; t_14_ = 11.6, p<0.0001) compared to water consumption by control rats (21.9±0.7 ml/day). No differences were found for the daily intake of food (saccharin group: 19.4±0.3 g/day; control group: 18.9±1.0 g/day) or calories (saccharin group: 56.3±0.9 Kcal/day; control group: 54.7±3.0 Kcal/day). Relative to the total energy intake, 2% of calories were derived from sugar for both saccharin and control groups. Body weight was also similar for the experimental groups (saccharin group: P30, 95.4±1.7 g and P46, 194.5±2.3 g; control group: P30, 94.0±4.4 g and P46, 191.0±8.5 g).

**Figure 5:**
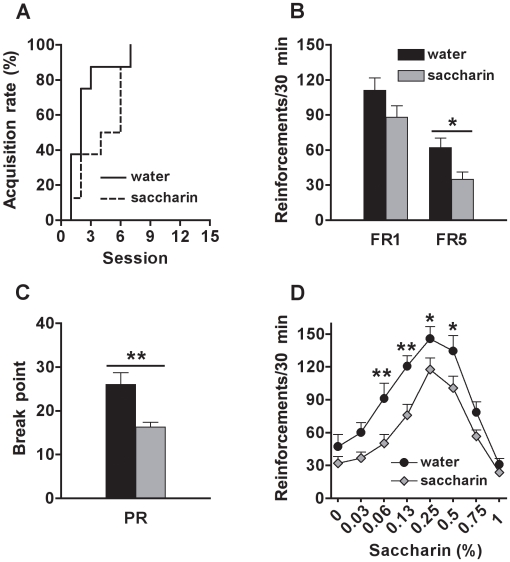
Motivation for saccharin (sweet) in adult rats exposed to saccharin or water during adolescence. (A) Percentage of rats reaching the acquisition criterion (>50 reinforcements for 2 consecutive sessions) for saccharin (0.13%, w/v) self-administration; (B) Number of saccharin (0.13%, w/v) reinforcements earned on fixed-ratio 1 (FR1) and fixed-ratio 5 (FR5) schedules; (C) Break point reached during the progressive-ratio (PR) test; and (D) Number of reinforcements earned (FR5) at different saccharin concentrations (in %, w/v), by adult rats exposed to saccharin (N = 8) or water (N = 8) during adolescence. *, ** Indicate significant group differences (p<0.05 and p<0.01, respectively).

The statistical analysis indicated that saccharin-exposed and control rats similarly acquired saccharin self-administration (n.s.) (Figure 5A). Although saccharin-exposed and control rats showed similar levels of saccharin intake during the FR1 (group effect: n.s.; session effect: F_(14, 196)_ = 16.5, p<0.0001; group x session interaction: n.s.), saccharin-exposed rats earned less saccharin reinforcements than control rats (group effect: F_(1, 14)_ = 6.6, p<0.05; session effect: F_(6, 84)_ = 14.2, p<0.00001; group x session interaction: n.s.) during the FR5 schedule (Figure 5B). Moreover, saccharin-exposed rats were less motivated for saccharin, these animals reaching lower break points during the PR test compared to the controls (group effect: F_(1, 14)_ = 10.5, p<0.01; session effect: F_(5, 70)_ = 3.7, p<0.01; group x session interaction: n.s.) (Figure 5C). During the concentration-response test, a bell-shaped curve was observed, with lower response levels at higher and lower concentrations of saccharin, and higher response levels for intermediate concentrations (concentration effect: F_(7, 98)_ = 76.9, p<0.0001). A group effect (F_(1, 14)_ = 6.3, p<0.05) and a concentration x group interaction (F_(7, 98)_ = 2.2, p<0.05) were also revealed by the ANOVA. The post-hoc comparisons indicated that saccharin-exposed rats showed lower levels of response for saccharin at 0.06% (p<0.01), 0.13% (p<0.01), 0.25% (p<0.05) and 0.5% (p<0.05) compared to the controls (Figure 5D).

## Discussion

We hypothesized that excessive activation of the brain reward system by sugar overconsumption during adolescence would alter reward function and lead to long-lasting behavioral changes. The results show that overconsumption of sucrose during adolescence (i.e., about 20% of total daily energy intake) leads to a considerable and sustained reduction in the motivation for both sweet and non-sweet solutions in adulthood. This enduring effect was specific for palatable drinks in that both the intake and motivation for cocaine were not affected by sugar overconsumption. Hence this effect is specific to adolescence since sugar overconsumption during adulthood only led to a modest and transient decrease in motivation for a sweet solution later in life. These later findings point to adolescence as the critical period for increased vulnerability to the sucrose effect.

Sugar overconsumption early in life induced a slower acquisition rate and lower intake of saccharin under a continuous schedule of reinforcement (FR1, FR5) in adulthood compared to rats without a history of sugar overconsumption. Like sucrose, saccharin activates sweet taste receptors [33], but is non-caloric. When the effort required to obtain the reinforcement was increased (progressive-ratio), rats that had unlimited access to sucrose during adolescence clearly showed a reduced motivation for saccharin compared to controls. These findings are consistent with a recent publication by Frazier et al. [12] reporting that 3-week old mice having unlimited access to sucrose pellets for 4 to 7 weeks display decreased motivation for sucrose in adulthood. It was also observed here that sugar overconsumption induced a clear downward shift of the concentration-response curve, however, the peak concentration for saccharin response was equivalent for both sugar-exposed and control rats, an effect indicative of changes in the reward function [34]. These changes appear to be independent of alterations in gustatory sensitivity, since any changes in this measure would have resulted in horizontal shifts to the right for decreased (tolerance) or left for increased (sensitization) gustatory sensitivity. Together, these results unambiguously show that sugar overconsumption during adolescence reduces the rewarding effectiveness of a sweet solution in adulthood.

Another point of interest was that the hedonic value of maltodextrin, a caloric non-sweet palatable substance [35], was also decreased by adolescent sugar overconsumption. Adolescent rats having free access to sucrose showed a slightly slower acquisition rate and overall decreased intake of maltodextrin during adulthood. No difference was found during the progressive-ratio test, probably because maltodextrin, though palatable, is a weaker reinforcer compared to saccharin (unpublished observations). In other words, the workload (price) for maltodextrin was too high to maintain the response (floor effect) in this test. However, adolescent sugar overconsumption produced a substantial downward shift of the concentration-response curve, further indicating changes in the reinforcement efficacy of maltodextrin. These results indicate that the effects of adolescent sugar overconsumption encompass broader phenomena in the brain reward function, which extend beyond the hedonic properties of sweet flavor.

The aforementioned effects of sugar overconsumption on motivation and reward function could be due to an unusually frequent and intense firing of brain gustatory reward systems via stimulation of peripheral sweet-taste receptors or, alternatively, the effect of post-ingestive (caloric load) consequences. However, the former seems more likely since the rewarding effect of sweet flavor, as indirectly measured by dopamine release in the *nucleus accumbens*, outweighs the rewarding effects of calories [36]. More importantly, adolescent overconsumption of a saccharin solution, which shows equal preference when compared to 5% sucrose (unpublished data), also reduced the motivation of rats for a sweet solution in adulthood. It is important to note here that the daily calorie intake was identical for rats with unlimited access to saccharin and control rats.

Cocaine self-administration was not affected by sugar overconsumption. These findings suggest the recruitment of alternative reward pathways associated with palatable drinks compared to those of cocaine. The lack of effect on cocaine self-administration was somewhat unexpected given the major role of the dopamine system in reward function and motivation for both cocaine and palatable drinks/foods [30], [31]. However, these results are in agreement with recent findings showing independent mechanisms of cocaine and food self-administration in mice lacking the dopamine transporter [37] and different neurobiological substrates for cocaine and food reward [38], [39]. Possible alternative pathways that may explain our results are changes in the endogenous opioid system following adolescent sucrose overconsumption. Indeed, it has been reported that knockout mice for endogenous opioids (beta-endorphin and enkephalins) show reduced motivation for both sweet and non-sweet natural rewards [40] and that opiate antagonists reduced sucrose craving [21] and the hedonic properties of palatable food [30]. Moreover, sugar overconsumption produces behavioral and neurochemical signs of opioid dependence [41], [42]. Together, these findings place endogenous opioids as a potential system implicated in the behavioral alterations observed in this study. Future studies will investigate the impact of sugar overconsumption during adolescence on heroin self-administration.

Interestingly, sugar overconsumption by young adult rats had only a moderate effect on saccharin self-administration later in adulthood. These rats showed similar acquisition rates and intake of saccharin during FR1 compared to control rats. Although there was a decreased self-administration of saccharin during an FR5 schedule in the adult sucrose-consuming rats, this effect was transient and no group differences were observed in the subsequent progressive-ratio and concentration-response tests. Therefore, our data suggest that adolescence is a period of high vulnerability to the enduring effects of sugar in adulthood. One possible reason for this effect could be the hyper-stimulation of the mesolimbic system by sugar overconsumption altering neural developmental processes (e.g., neuronal proliferation, migration, differentiation, synaptogenesis, apoptosis, etc) leading to enduring behavioral consequences.

A reduced motivation and reward function for palatable drinks or foods following sugar overconsumption might have a positive impact on health in certain disorders, such as bulimia, overeating or binge eating, which all present a component of increased hedonic eating [43], [44]. Conversely, lack of motivation and anhedonia are integral symptoms of depression [45], one of the most prevalent psychiatric disorders worldwide [46], schizophrenia, schizoaffective disorder, drug addiction, and eating disorders [45]. Recently, Moore and collaborators [47] have reported that the consumption of sweets (candy, confectionery) in childhood predicts convictions for violence in adulthood. Although it is difficult to establish a causal mechanism, this study reveals a largely overlooked relationship between sugar overconsumption early in life and psychosocial functioning in adulthood. It could be therefore suggested that overconsumption of sugar during critical periods of brain development, along with several other factors (stress, sedentary life, etc), contributes to the growing incidence of neuropsychiatric disorders worldwide [9], [48].

In conclusion, the results of this study show that overconsumption of sugar or no-calorie sweeteners during adolescence, but not adulthood, had an unsuspected period-specific long-lasting effect on motivation and reward function. Given our “highly sweet environment”, these findings may have implications in terms of public health policies, as altered motivation for natural rewards is a hallmark of several neuropsychiatric disorders whose incidence has escalated, as has sugar intake.

## Materials and Methods

### Ethics Statement

All experiments were carried out in accordance with institutional and international standards of care and use of laboratory animals [UK Animals (Scientific Procedures) Act, 1986; and associated guidelines; the European Communities Council Directive (86/609/EEC, 24 November 1986) and the French Directives concerning the use of laboratory animals (décret 87-848, 19 October 1987)]. All experiments have been approved by the Committee of the Veterinary Services Gironde, agreement number B-33-063-5, 13 June 2006.

### Subjects and Exposure to Sucrose during Adolescence

Male Wistar rats were purchased from Charles River Laboratories, France. Rats were individually housed in plastic cages and maintained under an inverted 12-h light/dark cycle (lights off at 8:00 h) at 21±2°C. Food (Scientific Animal Food & Engineering, France; containing 16.5% protein, 59% carbohydrate and 3% fat; with a caloric value of 2.9 Kcal/g) and water were provided *ad libitum* throughout, except during experimental sessions. After 4 days of acclimatization in our animal facilities, adolescent (post-natal [P] day 30) [7], [8] rats were given continuous access in their home cages to an additional bottle containing sucrose solution (5%, w/v) or water (controls) for 16 days. With this concentration of sucrose, the total daily energy intake coming from sugar (around 20%) displayed by adolescent rats approximates the sugar intake observed in human adolescents [5], [6]. The sucrose (or water) bottle was then removed and rats had access to water only. Animals were kept undisturbed in their home cages for two weeks before behavioral testing. To determine the age specificity in the reported effects, a complementary study was conducted with a similar procedure in adult rats exposed to sucrose from P60 to P76. Moreover, an additional group of adolescent rats was given continuous access to saccharin solution (0.25%, w/v), instead of 5% sucrose, in order to determine whether the reported effects of sucrose were due to its sweet taste or caloric load.

### Surgery

For cocaine self-administration, rats were anesthetized with an intraperitoneal injection of a mixture of Ketamine (100 mg/kg) and Xylazine (10 mg/kg) and surgically implanted with chronic intravenous Silastic catheters (Dow Corning, USA) into the right jugular vein. The catheter was secured to the vein with a suture thread and was passed subcutaneously to exit dorsally on the animals' back. After surgery, catheters were flushed daily with 0.2 ml of a sterile antibiotic solution containing heparinized saline (280 IU/ml; Sanofi-Synthelabo, France) and ampicilline (Panpharma, France). Rats were allowed to recover for seven days before behavioral testing.

### Apparatus

Self-administration sessions were conducted in standard operant chambers (30×40×36 cm; Imétronic, France) located in a dimly lit room with a background white noise (Imétronic, France). The chambers were individually enclosed in wooden cubicles fitted with a ventilation fan that also screened extraneous noise. Each operant chamber had two opaque panels as the right and left walls and two clear Plexiglas panels as the back and front walls. The floor consisted of 6-mm diameter steel bars spaced 15 mm apart. Two retractable levers (2×4×1 cm) were mounted 7 cm above the grid floor on the right operant panel. A white light diode was mounted 8.5 cm above each lever. A diffuse white light bulb (2 W) fixed on the top of the back wall illuminated the chamber. For cocaine self-administration, a spring-covered Tygon tube connected, through a fluid swivel, the animal's catheter to a syringe (placed outside the chamber) containing a cocaine solution. For saccharin and maltodextrin self-administration, a drinking reservoir was positioned 4 cm above the grid floor at equidistance between the two levers. The drinking cup was connected to a syringe (placed outside the chamber inside a syringe pump) that contained either saccharin or maltodextrin solution. A microcomputer controlled the delivery of fluids and presentation of visual stimuli and recorded behavioral data.

### Procedure

Solutions of saccharin (Sigma) and maltodextrin (Caloreen®) were used as sweet and non-sweet [33], [35] natural rewards, respectively. Cocaine (Coopérative Pharmaceutique Française, France), a potent drug of abuse that produces its reward effects through blockade of brain dopamine transporters [49], was used as a pharmacological reward.

All behavioral tests were conducted during the dark phase of the light/dark cycle, 5 to 7 days a week. Rats were trained to press one of the two levers (the active lever) on a fixed-ratio (FR) 1 schedule (each response resulted in fluid delivery) to obtain 0.11 ml (over 3 seconds) of either saccharin (0.13%, w/v) or maltodextrin (5%, w/v) in 30-minute daily sessions. Reinforced responses were followed by a 4 s time-out period, in which the cue-light (above the active lever) was turned on and lever presses did not result in additional fluid delivery. Presses on the other lever (inactive lever) had no programmed consequences. The positions of the active and inactive levers were counterbalanced in the operant chambers. For cocaine self-administration, rats received intravenous infusions of cocaine (250 µg/0.1 ml; over 3 seconds) followed by a 20 s time-out period (cue-light turned on) in daily 2 h sessions.

Rats were tested on FR1 for 15 (saccharin and maltodextrin) to 17 (cocaine) sessions and then shifted to an FR5 (5 lever presses resulted in fluid delivery) schedule for 6 (cocaine) to 10 (saccharin and maltodextrin) additional sessions. The criterion for acquisition of saccharin and maltodextrin was a minimum of 50 reinforcements per session over 2 consecutive sessions. For acquisition of cocaine self-administration the criterion was a minimum of 15 infusions per session over 2 consecutive sessions. Two rats from the sucrose group and 3 rats from the control group did not reach the acquisition criterion for cocaine self-administration and thus were excluded from the subsequent phases of the study.

Following training under the FR5 schedule, rats were tested under a progressive-ratio (PR) schedule for 5 daily sessions. In this test, the number of lever presses required to earn the next reward increased progressively by 3, starting at 1 (1, 4, 7, 10, etc). For each reinforced response, the animal received saccharin (0.13%, 0.33 ml), maltodextrin (5%, 0.33 ml) or cocaine (250 µg/0.1 ml). The breakpoint was defined as the last ratio completed before the response was disrupted. For saccharin and maltodextrin experiments, the session stopped after 2 hours or when 30 minutes elapsed without the rat receiving a reinforcer. For the cocaine experiments, the session stopped after 4 hours or when 35 minutes had elapsed without the rat receiving a cocaine infusion (i.e., 30+5 min that corresponds to the average inter-injection pause at the 250-µg dose). This five min-longer cut-off time was used for cocaine to take into account the “satiating” effect of the drug [50].

Following PR, rats were again maintained on FR5 for 3 sessions and then eight different concentrations of saccharin (1, 0.75, 0.5, 0.25, 0.13, 0.06, 0.03 and 0%; 0.11 ml), maltodextrin (40, 30, 20, 10, 5, 2.5, 1.25 and 0%; 0.11 ml) or cocaine (1000, 500, 250, 125, 62.5, 31.2, 15.6 and 0, µg/0.1 ml) were presented between-sessions in a descending order (one concentration per session).

### Statistical Analysis

All data are presented as means and standard error of the mean (SEM). Consummatory behavior and body weight were compared using the Student's t test. To compare the acquisition rate between the experimental groups, a Kaplan-Meier survival analysis was performed on the number of sessions required to reach criterium followed by a logrank test (GraphPad Prism version 4.03, GraphPad Software, USA). Each phase (FR1, FR5, PR and concentration-response curve) of the self-administration procedure was analyzed separately using analysis of variance (ANOVA) for repeated measures with group (sucrose, water) as a between-subject factor and session or concentration as within-subject factors. The Fischer's LSD test was used for post-hoc comparison of the means when appropriate (Statistica 9.0, StatSoft Inc., USA). The accepted level of significance for all tests was p<0.05.
